# Supplementary figures and images for: Evaluation of the Attractant Effect of Solanum muricatum (Solanales: Solanaceae) on Gravid Female Adults of Zeugodacus tau (Diptera: Tephritidae) and Screening of Attractant Volatiles
Source: Insects. 2023 Jun 30;14(7):591. doi: 10.3390/insects14070591 (PMC10380902; doi:10.3390/insects14070591)

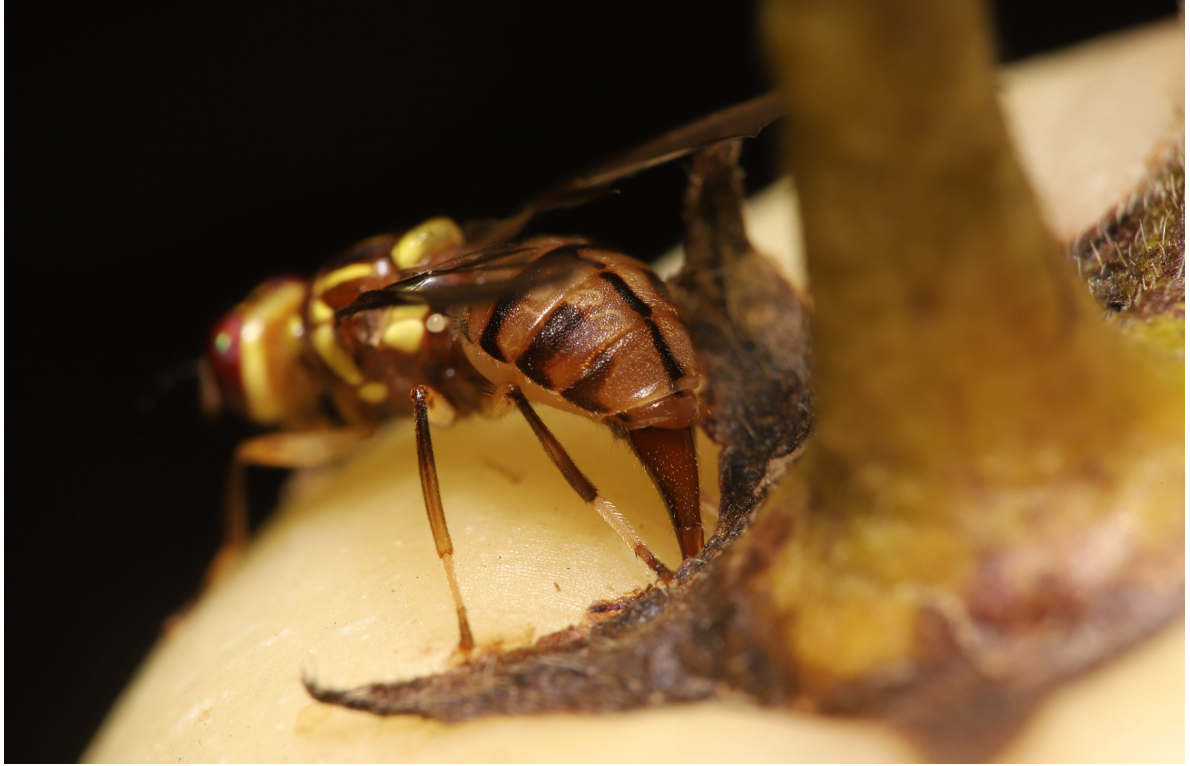

**Figure S1:** Ovipositing of *Z. tau* on *S. muricatum*.

Supplement: Supplementary file 1 [file insects-14-00591-s001.zip › insects-2432180-supplementary.pdf]
